# Localized microwave-heating (LMH) of basalt – Lava, dusty-plasma, and ball-lightning ejection by a “miniature volcano”

**DOI:** 10.1038/s41598-019-49049-5

**Published:** 2019-09-10

**Authors:** Eli Jerby, Yoav Shoshani

**Affiliations:** Faculty of Engineering, Tel Aviv University, Ramat Aviv, 6997801 Israel

**Keywords:** Volcanology, Electrical and electronic engineering, Plasma physics

## Abstract

This paper presents various phenomena obtained by localized microwave-heating (LMH) of basalt, including effects of inner core melting, lava eruption and flow (from the molten core outside), plasma ejection from basalt (in forms of fire-column and ball-lightning), and effusion of dust (deposited as powder by the plasma). The experiments are conducted by irradiating a basalt stone (~30-cm^3^ volume, either naturally shaped or cut to a cubic brick) in a microwave cavity, fed by an adaptively-matched magnetron (~1 kW at 2.45 GHz). Effects of LMH and thermal-runaway instability in basalt are observed and compared to theory. Various *in-* and *ex-situ* diagnostics are used in order to characterize the dusty-plasma observed and its nanoparticle products. The resemblance of the experimental phenomena obtained in small laboratory scale to gigantic volcanic phenomena in nature is noticed, and its potential relevance to further volcanic studies is discussed. In particular, we show that LMH could be instrumental for laboratory demonstrations and simulations of miniature-volcano effects, such as lava flows, formation of volcanic glass (obsidian), eruption of dusty-plasma and volcanic ash, and ejection of ball lightning. These findings might be significant as well for various applications, such as drilling and mining, microwave-induced breakdown spectroscopy (MIBS), mineral extraction, and powder production directly from basalts.

## Introduction

Microwave heating is unique, compared to other heating techniques, because it may initially generate the thermal energy inside the irradiated object (due to the penetration of the microwave radiation inside, and its absorption and conversion to heat therein)^1^. Furthermore, localized microwave-heating (LMH) and the consequent formation of hotspots by thermal-runaway instability may cause local melting in various materials (e.g. silicates^2^), and even plasma ejection outside.

The thermal-runaway instability^3^, undesired in most microwave applications, may intensify the heat localization effect^4,5^. It occurs in materials with temperature-dependent properties, which cause the hotter region (typically the core) to become more susceptible to further microwave heating than the outer, cooler parts. This positive-feedback instability localizes the microwave heating and concentrates the thermal energy, and hence eventually leads to creating of a molten hotspot.

**Figure 1:**
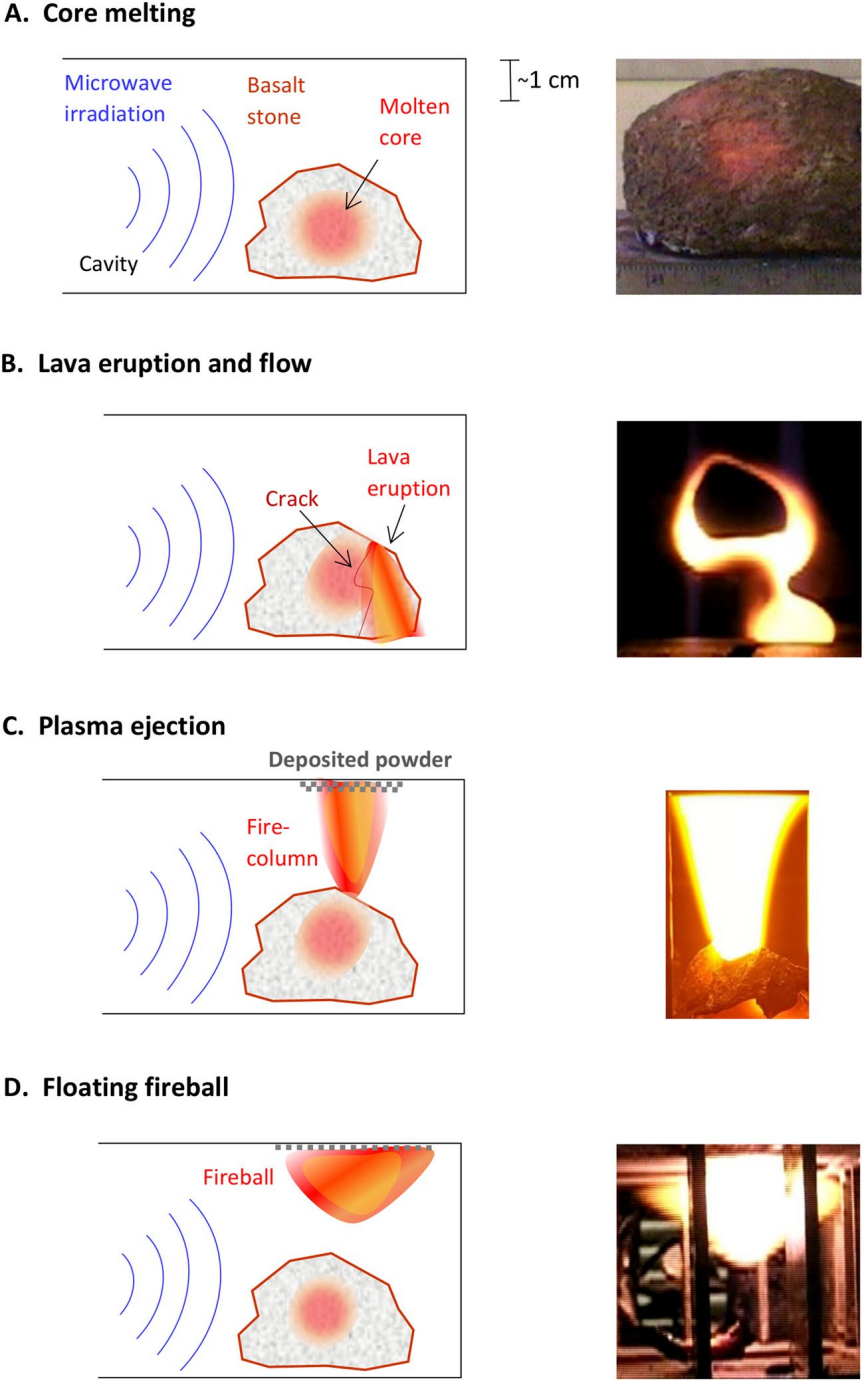
Conceptual illustrations of the LMH process in basalt. (**A**) Core melting – Microwave energy is irradiated into the core of the basalt stone, converted to heat inside, and melting the core by an LMH mechanism. The molten core (seen in the image as an inner magma via the porous solid surface) can be maintained stably in equilibrium. (**B**) Lava eruption – A slight increase in the microwave power may elevate the pressure inside the stone and induce cracks from which the lava erupts and flows out, onto the basalt surface. The image shows the molten core pouring out like volcanic lava streams. (**C**) Dusty-plasma ejection – Further irradiation causes a plasma emission from the molten basalt. The image shows a plasma fire-column ejected upward from the molten basalt. (**D**) Floating fireball – A ball-lightning-like fireball is evolved from the plasma plume (either associated with a fire-column or alone). The image shows a fireball hovering inside the cavity.

Microwave-heating effects in basalts, such as melting, cracking and crushing^6–12^, have been studied in various contexts mainly related to mining and construction applications. The thermal-runaway effect in basalt^5,9^ leads to a hotspot formation and melt inside the stone^6,9,10^, as illustrated in Fig. 1A. Further irradiation of the basalt stone by a regulated microwave power can stably maintain the molten core inside, in a thermal equilibrium. However, a slight increase in the power may elevate the temperature and increase the pressure inside the stone. Cracks may appear then on the rock surface; the inner melt may erupt outwards and flow as a lava stream over the surface (Fig. 1B), like a “miniature volcano”. Here we have also found that a further irradiation of the basalt heats the hotspot up to a point in which plasma is ejected from its surface, as shown in Fig. 1C ^13^.

Plasmas are usually generated from basalts by various other means, not involving microwaves. The experimental studies reported in the literature include, for instance, plasma generated from basalt by CO2 and Nd:YAG lasers (for laser-induced breakdown spectroscopy, LIBS)^14^; inductively-coupled plasmas of basalt in various forms (rock, fiber and glass) analyzed by atomic emission spectroscopy^15^; and a plasma spray of basalt in powder form implemented in a basalt coating technique^16^.

As observed in the present work^13^, starting from an LMH hotspot in basalt, the plasma is first ejected and evolves as a fire-column, which is stably maintained by the microwave irradiation. Then, it may also emit a fireball, buoyant in the air atmosphere within the cavity, as shown in Fig. 1D. This basalt-originated fireball is either adjacent to the initiating fire-column or detached from it, tending to hover towards the microwave source (as in refs^2,17–19^).

This paper presents observations of volcano-like phenomena in basalt-LMH experiments, also in comparison to theory; characterizes the dusty-plasma ejected and its nano-powder products; and discusses the LMH potential as a means for experimental laboratory-scale simulations of volcanic effects, and for other material-processing applications.

## Experimental Setup

**Figure 2:**
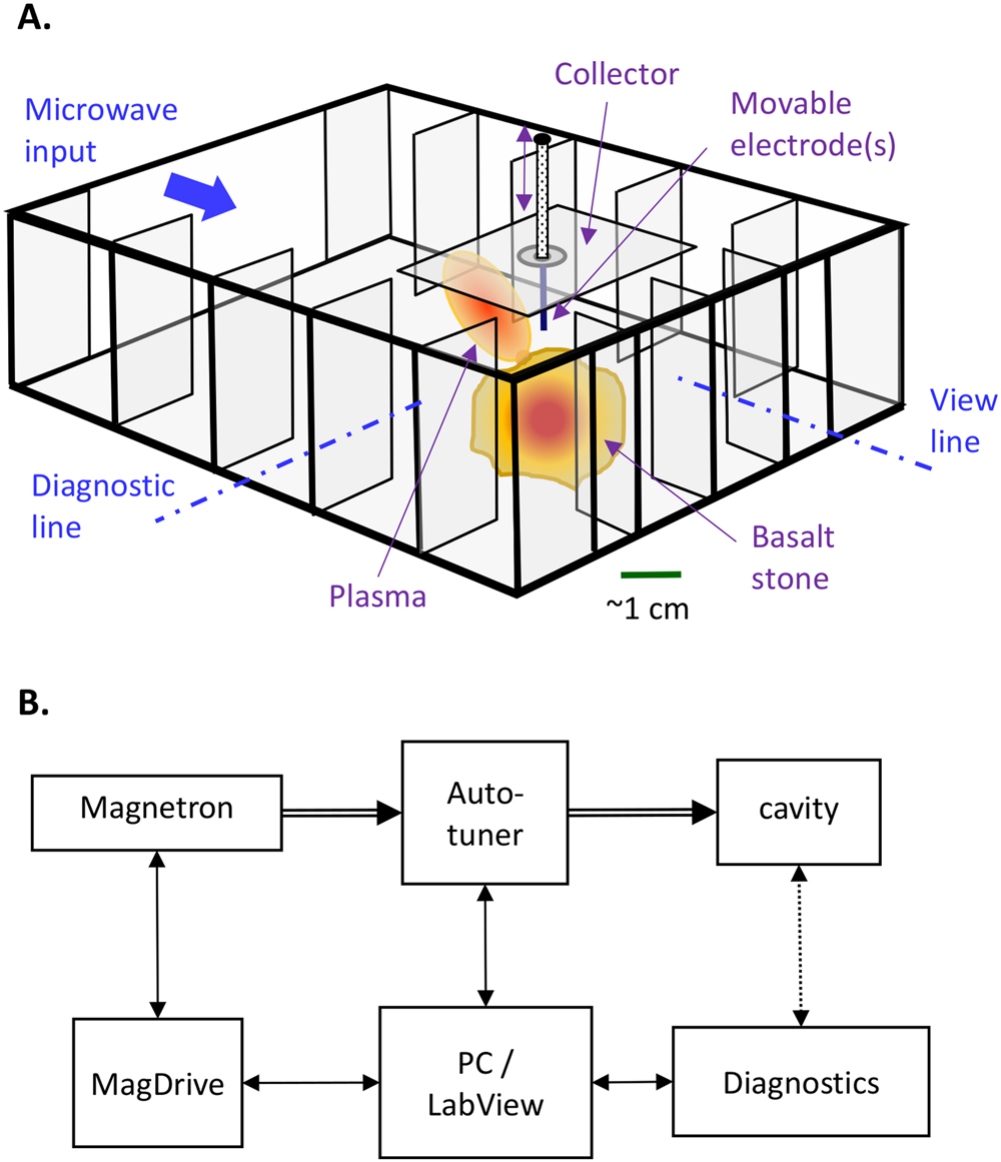
The experimental setup employed. (**A**) The microwave cavity in which the basalt stone (or a cubical brick) is situated and subjected to the LMH interaction. The sidewalls consist of periodic structures under cutoff, which prevent microwave leaks but enable an open access to the interaction (e.g. for a real-time diagnostic with a direct line-of-sight). A movable electrode (or an electrode array) may optionally direct the microwaves into the basalt core, and hence expedites the creation of the molten hotspot inside. By tuning the microwave power, lava can be erupted from the molten core. Furthermore, a plasma column (and even a fireball) can be ejected upward to the air atmosphere, and deposit nanoparticles onto the collector (situated on the chamber ceiling). (**B**) A block diagram of the experimental instrumentation. The typical diagnostics include an optical spectrometer, thermal and video cameras, microwave scattering measurements, and an *I*–*V* probe.

The LMH experiments presented in this paper have been conducted in the experimental setup illustrated in Fig. 2A,B [Method 1]. The microwave cavity (Fig. 2A) is made of a 10 × 5 cm^2^ cross-section waveguide, shorted at the end by a microwave reflector. This cage-like structure of the cavity, with wide openings (~22 mm) between the vanes, enables direct access and broad view from various directions into the interaction region (with immediate lines of sight for the various diagnostic means). The cavity is fed by a 2.45-GHz magnetron (regulated up to 1-kW input power).

By applying LMH, the inner core of the basalt is first melted^6,8–10^. Further irradiation leads to the eruption of the molten basalt, from the core to the surface (similarly to lava eruption and flow in volcanoes). The molten basalt cools down and solidifies to a black glass (obsidian). A further irradiation causes the basalt to emit a plume of dusty plasma, which evolves to a confined plasma column, and further to a plasmoid similar to a natural ball-lightning. These fire-column and fireball-like plasmoids are characterized here by various *in-* and *ex-situ* means.

The optional use of a movable electrode (as in the microwave-drill device^20^ and in previous plasma studies^2,21^) may expedite the eruption process by intensifying the local electric field. However, similar effects may occur in basalt with no electrode as demonstrated here as well (though in a less controllable fashion). The electrode enables reaching the melting temperature more locally and rapidly within a hotspot underneath the surface, which provides a path for lava eruption from the molten core. Here we also examined the use of an electrode array in order to induce multiple hotspots.

## Results

### Core melting and lava eruption

**Figure 3:**
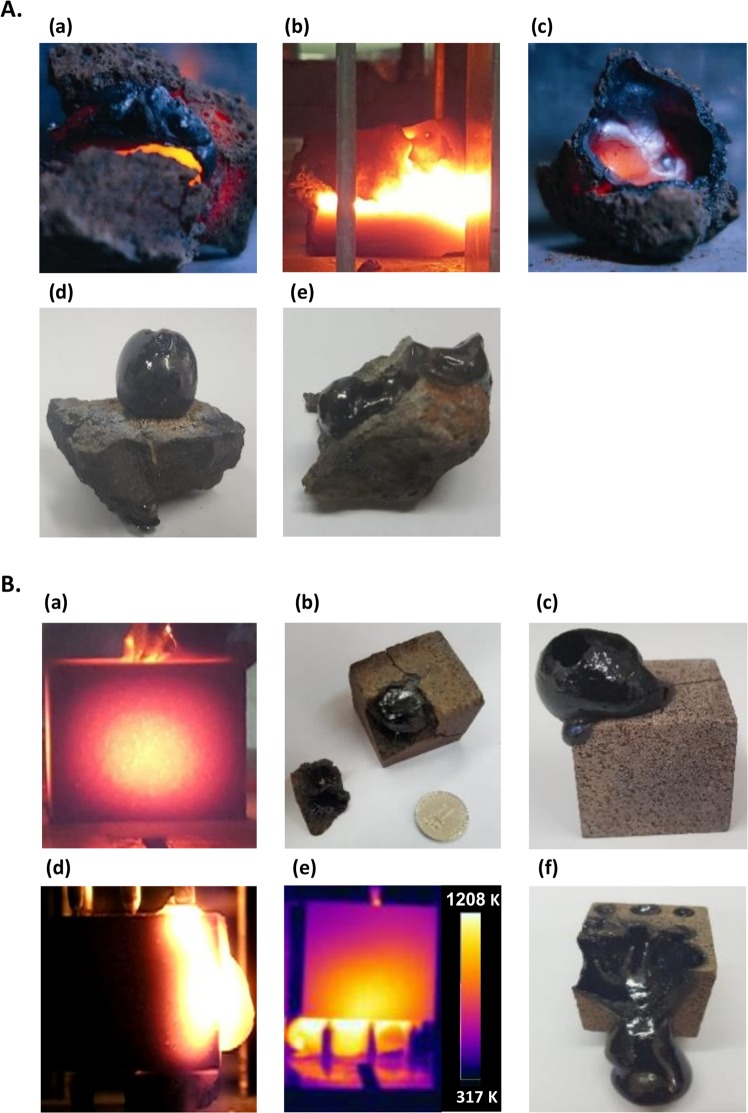
Basalt heating and melting by LMH, lava flows and vitrification to obsidian. (**A**) Naturally-shaped basalt stones (of ~5-cm length) under LMH: (a) Inner core melting; (b) lava eruption and flow; (c) formation of a crater; (d) a dome of a thin layer of volcanic glass (obsidian) created by lava bubbling; (e) a trace of lava stream solidified to obsidian. (**B**) Electrode-enhanced LMH interactions with basalt stones cut to 3-cm side cubes (this well-defined geometry enables a better control of the experimental repetition, and a more precise comparison to numerical simulations): (a) An image of the inner hotspot captured via the solid outer side of the cube; (b) a basalt cube after a partial LMH interaction (stopped before eruption) revealing the molten core inside which has been solidified to obsidian. The outer surface has remained in its original texture except some cracks (the corner was removed in order to expose the molten inside)); (c) a bubble dome blown up during the lava eruption (made of a thin layer, crispy and fragile, of obsidian); (d) lava flows from the cube’s molten core to its outside; (e) a thermal image of lava eruption from the core through multiple hotspots in the bottom surface induced by an electrode array (the lava temperature exceeds 1,200 K); (f) a hollow core remains after the lava has erupted and streamed outside, leaving a void inside and a solidified traces of obsidian.

Effects of core melting, followed by lava eruption and flow (and further solidification to obsidian) have been observed in LMH of natural basalt stones in these experiments, as shown in Fig. 3A (with no electrode). As an alternative to the naturally-shaped stones, cubic bricks (of 3-cm side length) cut from natural basalts have also been experimented, since their well-defined geometry enables better comparison with numerical simulations. The LMH experiments with basalt cubes (using electrodes) resulted in similar effects of core melting, and lava eruption and flows, as shown in Fig. 3B. The thermal image in Fig. 3B(e) indicates that the molten lava temperature exceeded ~1,200 K. The lava erupts from the core outwards, and then flows downwards by gravity with a front-flow velocity of ~1 cm/s, while its color varies from luminous white to orange and red [Supplementary Video S1a and S1b]. It eventually cools down and vitrifies to black volcanic glass, namely obsidian. Various other volcano-like phenomena are observed (as also shown in Fig. 3A,B), such as formations of bubbles and domes, fissures and craters, vents, blasts, bursts of lava, and lava tunneling. The latter is observed for instance when two hotspots are simultaneously created by electrodes in two opposite faces of the basalt brick. An inner lava tunneling effect is evolved then, in the core between the two hotspots, as observed via the porous outer surface of the basalt brick [Supplementary Video S2].

**Figure 4:**
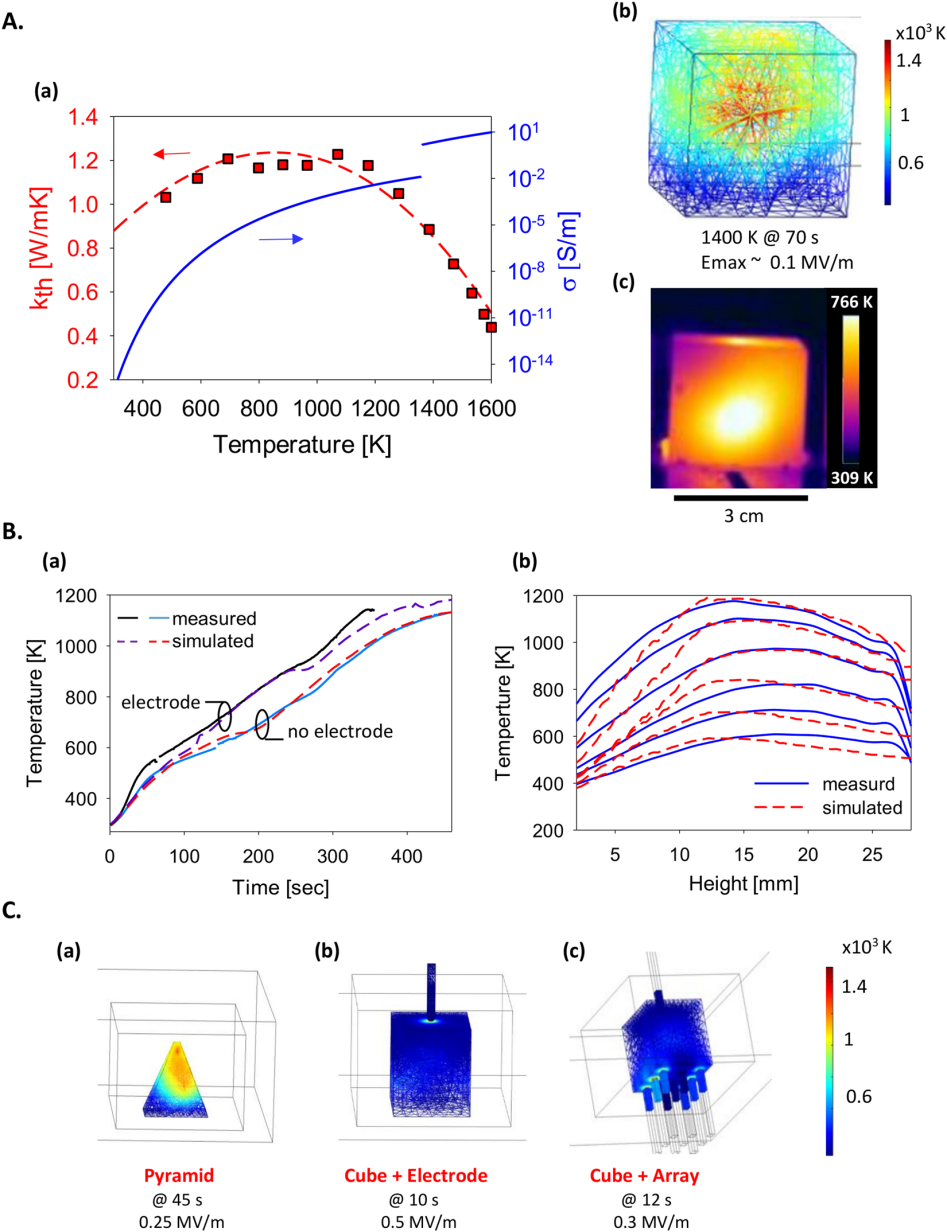
(**A**) LMH of a basalt cubical brick (with no electrode) and the temperature profile evolved: (a) The temperature dependencies of the thermal and electric conductivities, *k*_*th*_ and *σ*_*c*_, respectively, which enable the basalt LMH. (b) A numerical simulation of the basalt LMH process [Method 2] shows the molten core inside the irradiated basalt cube, reaching 1,400 K after 70 s, with no electrode. (c) A thermal image of the molten core captured via the solid outer sides of the cube (note also the temperature profile on the upper surface). (**B**) Comparison between numerical simulation and experimental results of the temporal and spatial evolution of the temperature profiles on the basalt cube surface: (a) The temporal evolution of the temperature on the face center, with and without an electrode; (b) the spatial temperature evolution along the outer face surface of the basalt cube with no electrode (the time intervals between curves correspond to the no-electrode curve in Fig. 4B(a)). (**C**) Numerical simulations of various shapes intended to intensify the LMH process as compared to the no-electrode scheme simulated in Fig. 4A(b) at the same input power: (a) A pyramid stone shape; (b) a single electrode, and (c) an electrode array. The most rapid surface melting is obtained with a single electrode (0.5 MV/m in 10 s) whereas the no-electrode scheme provides an inner core melting, as shown in Fig. 4A(b,c).

The theoretical analysis of the basalt’s LMH interaction ([Method 2] incorporates the thermal-runaway instability and the interactive temperature-dependence of the basalt’s properties^9^. The numerical simulations presented in Fig. 4A–C show the inner hotspot formation and core melting prior to the lava eruption, in agreement with the experimental results (with no electrode). The additional effect of the sharp geometry on the LMH evolution is demonstrated in Fig. 4C(a–c), by simulations of various shapes such as a sharp stone (a) and single- and multiple-electrode schemes (b, c).

### Dusty-plasma ejection in forms of fire-columns and fireballs

**Figure 5:**
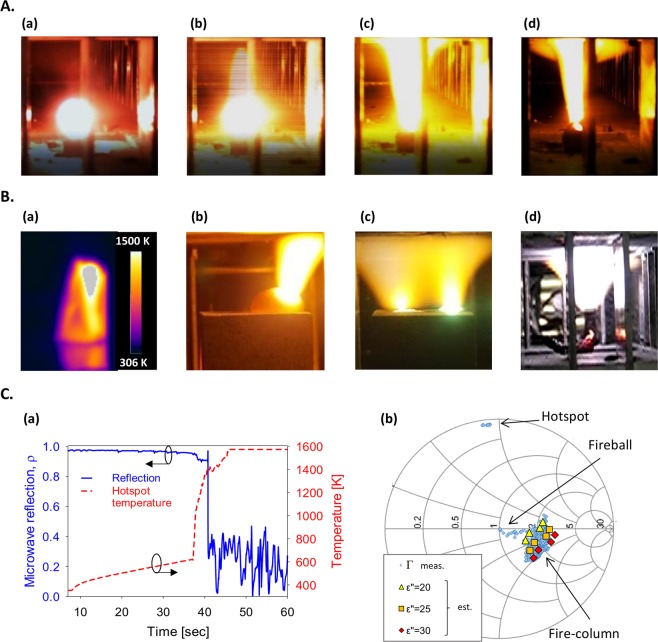
Plasma ejection from molten basalts in fire-column and fireball shapes. (**A**) The plasma evolution from a molten hotspot in naturally-shaped basalt stone: (a) The hotspot is formed and further evolved; (b) a plasma plume is initially ejected from the hotspot; (c) a stable fire-column is fed by the hotspot emission; (d) a fireball is separated from the fire-column (beyond it), and hovers in the air atmosphere. (**B**) Various observations of plasma ejection from molten basalts: (a) A thermal image of a hotspot with a temperature exceeding 1,500 K, which emits plasma; (b) a plasma column ejected from a dome of molten basalt, blown up by the lava eruption from the core; (c) a pair of plasma columns ejected from two adjacent hotspots evolved on the basalt surface; and (d) a stand-alone fireball floating in the air atmosphere within the cavity. (**C**) Microwave reflections during the various stages of the plasmoid evolution: (a) The reflection coefficient *ρ* = |Γ| and the hotspot temperature measured vs. time, showing an abrupt drop of the microwave reflections during the thermal-runaway instability rise. (b) A Smith-chart presentation of the complex reflection coefficient Γ, starting from a mismatch (|Γ| ~ 1) at the hotspot stage (as in Fig. 5A(a)), reduced to |Γ| ~ 0.4 at the fire-column stage (Fig. 5A(c)), and matched to |Γ| ~ 0 by the self-adapted fireball (Fig. 5A(d)). Simulation results of dielectric dummy loads (of ${\varepsilon }_{r}^{^{\prime\prime} }$ = 20, 25, 30) with a similar column shape in various positions, are shown for comparison in order to estimate the effective plasma impedance.

In this study we have found that a further microwave irradiation of the molten basalt causes a plasma ejection from it. In this process, the microwave localization effect continues to evolve and to heat up the melt, and hence plasma is ejected from it as shown in Fig. 5A(a–d) and Supplementary Video S3. Starting from a molten hotspot (a), the plasma is first emitted (b), and then forms a stable fire-column (c). In some cases, another plasmoid is also evolved in a form of a fireball, either alongside the fire-column (d) or detached from it as shown in Fig. 5B(d) and Supplementary Video S4. The detached fireball is typically less stable than the fireball adjacent to a fire-column, and it tends to move towards the magnetron. The fireball disappears within a few seconds, similarly to natural ball-lightning^22^. Other modes of plasma ejection from basalts observed in this study are a fire-column eruption from a dome made of a thin layer of obsidian (blown up by the lava eruption), and a simultaneous ejection of two plasma columns from two different hotspots, as shown in Fig. 5B(b,c), respectively (both effects are presented in Supplementary Video S5).

### LMH and plasma characterization

Various diagnostic techniques were applied in order to characterize the LMH of basalt, as well as the plasma ejected from it, and its products. These diagnostic means include microwave-scattering analyses, optical spectroscopy, and scanning electron microscopy (SEM). The analyses of the microwaves scattered from the plasmoid results in an estimate of its microwave impedance, which provides a measure for its dielectric and plasma properties. The reflections of the 2.45-GHz microwave input are detected during the various stages of the LMH process. The complex reflection coefficient Γ (Fig. 5C(a)), also presented in a Smith-chart form in Fig. 5C(b), shows that the microwave reflection tends to decrease (from |Γ| ~ 1 to ~0.4) after the plasma ejection, and to further decrease (to |Γ| ~ 0) when a fireball emerges (Supplementary Video S6). At the stable fire-column stage, the reflection coefficient is bouncing around ~0.4, while being attracted by the Smith-chart origin. The corresponding power reflection is smaller than 20% at this stage, hence the plasmoid interacts with its microwave source as an adaptively matched load. At the fireball stage, this self-tuning mechanism tends to maximize the microwave power absorbed by the fireball, by optimizing its intensity, position and size. The estimate of the plasma impedance and dielectric properties is based on a comparison of the reflection measurements with simulation results of a dummy load, with equivalent shape, position and size^18,19,23^.

**Figure 6:**
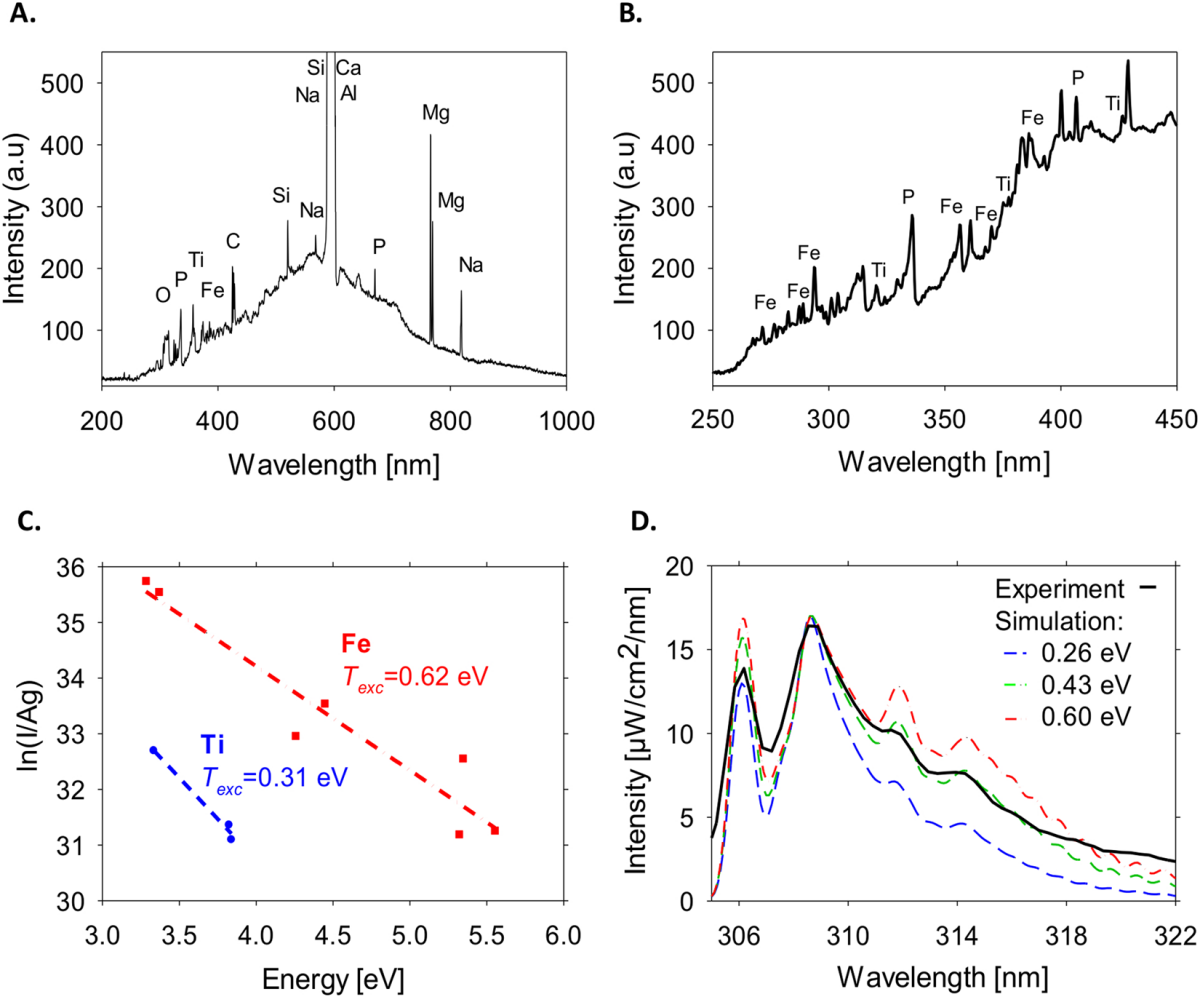
Optical spectroscopy measurements and analyses of basalt plasmoids: (**A**) A typical spectrum of atomic emission lines detected in the basalt’s plasma. (**B**) The spectrum observed in the short-wavelength range. (**C**) A Boltzmann plot of the Fe and Ti lines displayed in Fig. 6B above. (**D**) The OH-radical emission compared (by fitting) to LifBase simulation, both result in temperature estimates in the range *T*_exc_ ~ *T*_rot_ ~ 0.3–0.6 eV.

The optical spectrum of the light emitted by the basalt plasma-column (Fig. 6A,B) reveals its atomic lines, which are identified using^24^ as of Fe, P, C, Si, Al, Mg, Ti, and Ca. All these elements are known as ingredients of basalt rocks. The Boltzmann plot technique, implemented on the Fe and Ti lines, results in excitation temperature estimates of *T*_exc_ ~ 0.3 and ~0.6 eV, respectively (Fig. 6C). The spectral emission of OH radicals is identified by fitting to LifBase data^25^. The rotational temperature is also roughly estimated in the same range of *T*_rot_ ~ 0.3–0.6 eV (Fig. 6D).

**Figure 7:**
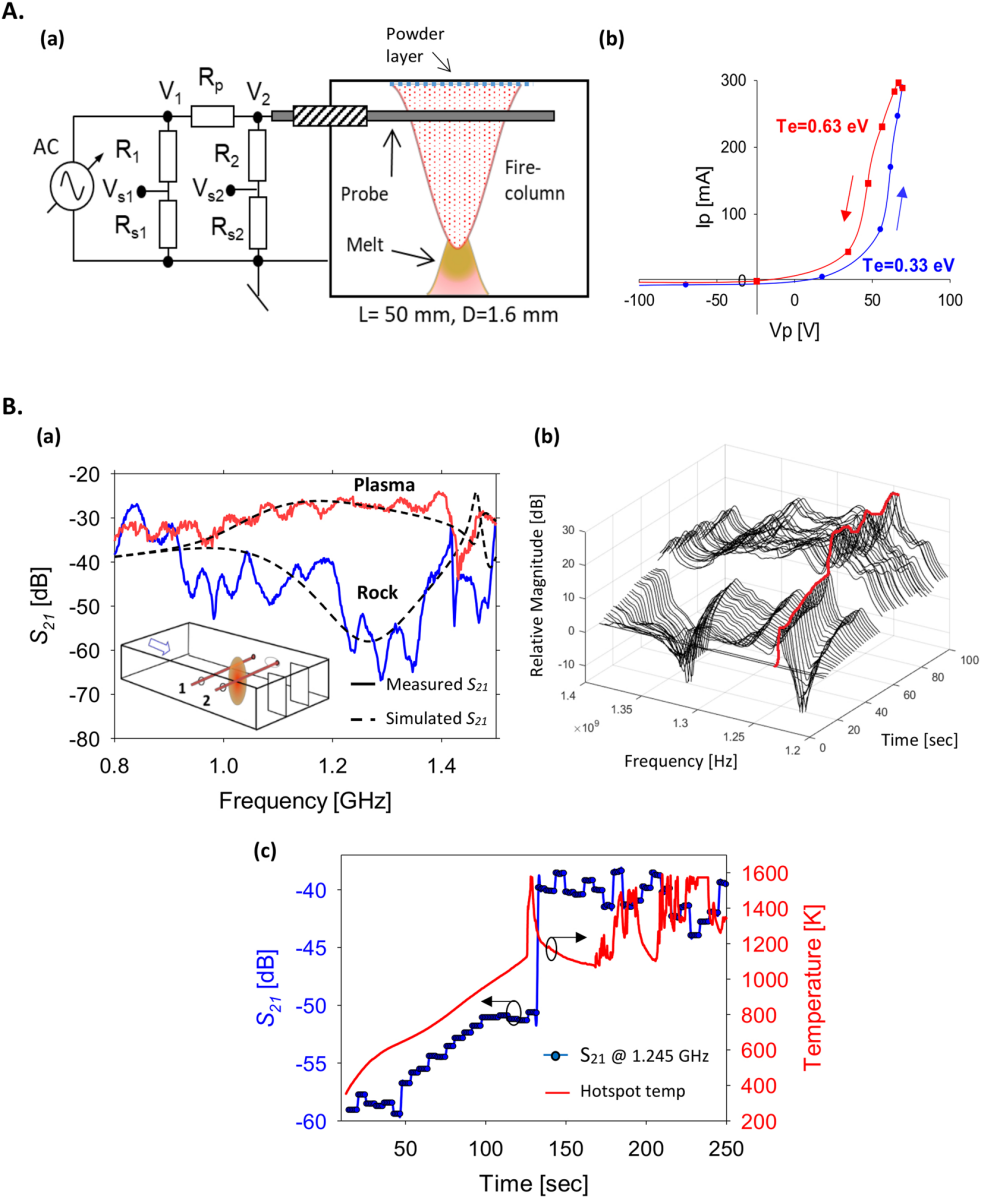
(**A**) Characteristic *I-V* curve measurements: (a) The electrical setup of a Langmuir-like probe fed by a 50-Hz, 100-V alternating voltage, and (b) a typical *I-V* curve measured with this probe. The different voltage rise and fall paths (arrowed) are attributed to the additional capacitance created by the powder deposition by the plasma on the probe. The electron temperature estimated is ~0.3–0.6 eV, similarly to the optical measurements above. Such *I-V* curves are only obtained when the melt flows down, as illustrated in Fig. 7A(a), and closes the electric current loop to the grounded aluminum floor (note that the powder deposited by the plasma isolates the upper collector plate). (**B**) Scattering analyses of the plasma by an additional probing-signal sweep at the frequency range 0.8–1.5 GHz: (a) The transmission coefficient *S*_21_, measured with and without plasma, shows a > 20 dB increase in the coupling due to the plasma shortening of the coupler arms 1 and 2 (shown in the inset). An equivalent load simulation results in *ε*_*r*_ ~ 0.3 − *j*50 for this effect. (b) The *S*_21_ spectral variation vs. time during the plasma evolution, and (c) the *S*_21_ transmission variation vs. time at the anti-node frequency (~1.245 GHz), with respect to the hotspot temperature variation. The abrupt increase in the transmission coefficient is associated with the decrease in the measured hotspot temperature, which occurs at the plasma ejection.

A Langmuir probe is inserted into the plasma in order to measure its *I-V* characteristics (Fig. 7A(a)). Valid results are only observed when the melt flows to the grounded chamber floor, and closes the electric circuits to enable a current loop (otherwise, smaller currents are measured due to the high impedance of the powder layer deposited by the plasma on the ceiling) [Supplementary Video S7]. The hysteresis observed in the *I-V* curve (Fig. 7A(b)) is attributed to the capacitive effect created by the powder deposited on the electrode. A rough estimate of the electron temperature by the curves’ slopes results in *T*_e_ ~ 0.3 eV and ~0.6 eV, for the voltage rise and fall, respectively. This range coincides with the results estimated by optical spectroscopy.

Microwave scattering measurements are also performed at other frequency ranges, by irradiating the plasmoid by another probing signal at low power. The latter is coupled via additional input and output ports (marked 1 and 2, respectively, in the inset of Fig. 7B(a)). Scattering results for the microwave transmission coefficients (measured and simulated) at a lower frequency band (0.8–1.5 GHz) are shown in Fig. 7B(a), with and without the plasma. The results show that near the anti-node frequency of the coupling between Ports 1 and 2 (~1.25 GHz, corresponding to *L* ~ λ/2, where *L* is the coupler arm length), the transmission coefficient *S*_21_ is increased by >20 dB in the presence of plasma. Indeed, below the plasma frequency, the fire-column behaves like a conductor, which shorts the coupler arm length by half (~*L*/2 ~ λ/4) and hence significantly increases the microwave transmission between Ports 1 and 2. A numerical simulation of this coupler, with an equivalent column-shaped load inside, enables us to estimate the plasma dielectric permittivity. As shown in Fig. 7B(b,c), the transmission increase at the anti-node frequency follows the abrupt temperature rise, in which the plasma switches from the decoupled to the coupled state of Ports 1 and 2.

### Dust effusion and *ex-situ* analyses

**Figure 8:**
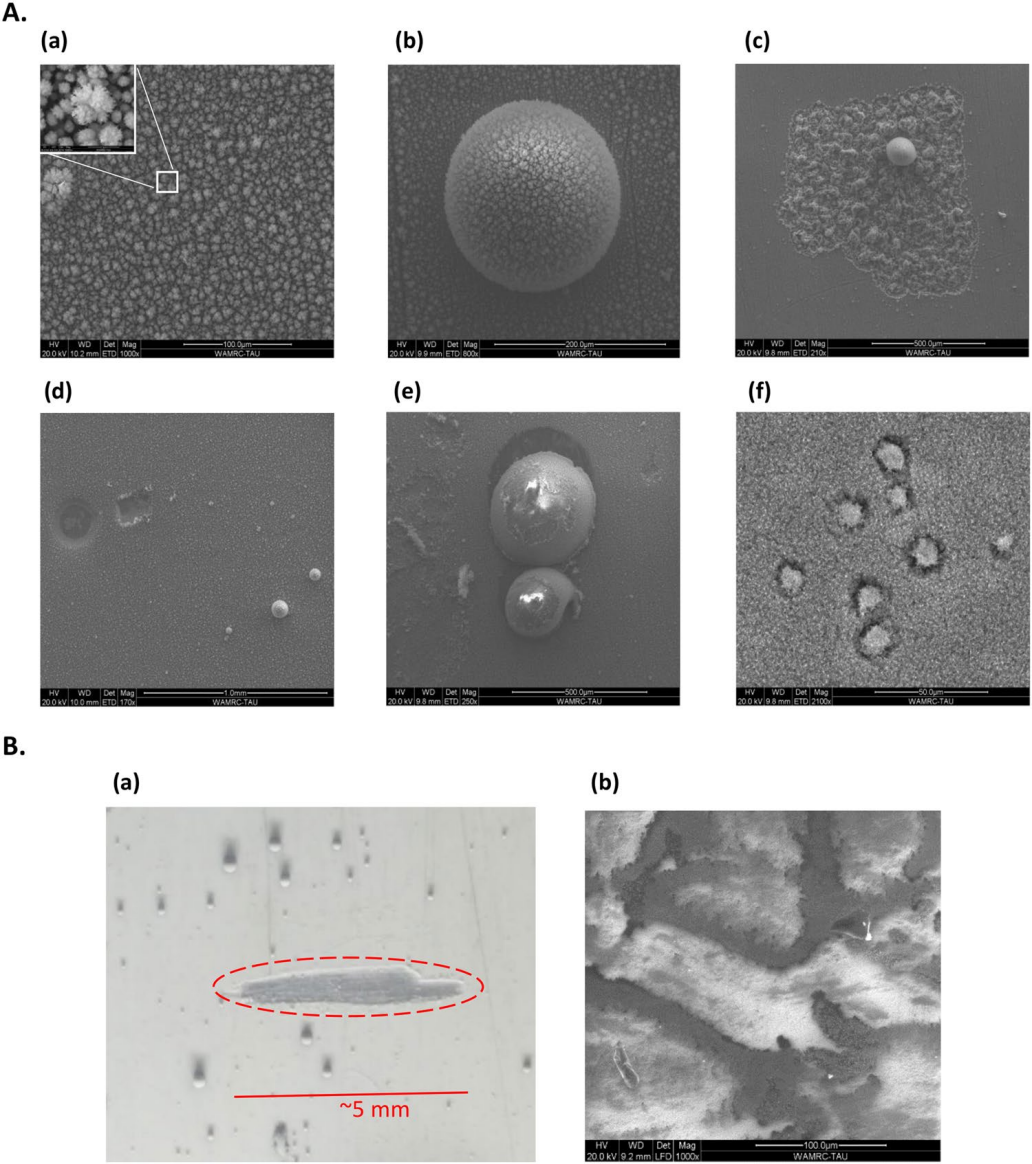
*Ex-situ* SEM observations of the dust products deposited by the plasma. (**A**) SEM observations of the collector: (a) Aggregates of flower-like shapes uniformly spread over the collector surface. The inset shows a typical particle of a ~5-*μ*m diameter; (b) an example of a larger sphere observed, of a ~ 0.2-mm diameter; (c) an island-like region, mostly consists of aluminum (possibly due to plasma etching of the collector); (d) craters and holes of comparable sizes of ~0.1-mm diameter; (e) spherical particles reside in craters, which could be attributed either to particle bombardment or to a plasma localization effect; (f) the collector surface covered by aggregates of flower-like shapes spread over it as a layer, with several traces of larger structures (~20-*μ*m diameter) immersed in it. (**B**) (a) An optical image of the white powder accumulated on the collector, with relatively large spherical particles observed. The deposited powder layer thickness measured by SEM is 10–30 μm thick (the scratch marked in Red was made in order to evaluate the layer thickness). (b) A SEM image of the volcanic glass obtained shows stream-like darker structures in the vitrified obsidian, and brighter island-like areas in between. EDS analyses of this glossy products result in the elemental composition presented for obsidian in Table 1.

A significant amount of dust is produced by the basalt’s plasma, deposited as a white powder on the collector shown in Fig. 2A. The surface morphologies of the particles created by the plasma are characterized by a scanning electron microscope (SEM) [Method 1]. The accumulated particles are observed in various shapes, mostly aggregates in few micro-meter sizes. Figure 8A(a–f) show aggregates of such particles on the collector surface as observed by SEM. Analyses of energy dispersive spectroscopy (EDS) reveal that these aggregates are composed of the elements listed in Table 1, also typical to the original basalt content. In addition, the basalt’s plasma generates larger spherical particles, in sizes of tens of microns and larger, as seen on the collector (Fig. 8A,B(a)). Holes of similar sizes are also noticed on the collector surface (this finding may either hint particle bombardment or a plasma localization effect). Island-like regions are observed upon the white powder deposition on the collector, as seen in Fig. 8A(c). EDS shows that they mostly consist of aluminum, as the collector itself, hence they may indicate an etching effect.

**Table 1:** The elemental composition of the original basalt stone and the two solid phases produced; the vitrified melt (obsidian) and the powder accumulated on the collector (typical values in Wt %).

	O	Si	Al	Fe	Ca	Na	Mg	K	Ti	P
Basalt	48	23	8	7	6	3	2	1	1	1
Obsidian	52	21	6	6	6	2	5	<1	1	<1
Powder	42	4	5	3	—	22	—	7	—	17

EDS analyses were also conducted on the basalt stone in its original form and its molten state (Fig. 8B(b)), in order to compare the compositions of the vitrified product (obsidian) and the natural stone. Table 1 shows the relative presence of the constitutive elements in the various phases of this process, namely the natural stone, the melt, and the deposited powder. The weight ratios differ between the various phases. However, it seems that the deposited powder consists of elements provided solely by the basalt (e.g. Si and Fe). These ingredients of the powder were most likely extracted from the basalt, and then evolved and transported by the plasma to the collector. Consequently, these elements were also detected within the plasma by optical spectrometry.

X-ray diffraction (XRD) pattern of massive samples of the molten basalt show low intensity of diffraction peaks, suggesting that the great part of the sample consists of glass. From the position of the peaks, it is possible to determine that the two major crystalline phases are an oxide of aluminum, magnesium and iron, and a silicate of magnesium and iron. The diffraction pattern of the white deposition (Fig. 8B(a)) enables to determine the presence of crystalline phases in the powder, including three silicate phases (Enstatite (Mg,Fe)_2_Si_2_O_6_, Augite (Ca,Mg,Fe)_2_Si_2_O_6_ and Mullite 3Al_2_O_3_·2SiO_2_) and two oxides (Periclase MgO and Wustite Fe_0.9_O). Peak observed are related to a phase with phosphor, possibly in amorphous phase (as confirmed by the modulated background).

A simplified model of microwave interaction with dusty plasma^26^ [Method 3] yields an estimate for the electron density in the basalt-originated plasma as *n*_*e*_ ~ 10^17^–10^18^ m^−3^, assuming an electron temperature of *T*_*e*_ ~ 0.3–0.6 eV (estimated by the various measurements above), and dust number density of *n*_*d*_ ~ 10^16^ m^−3^, as measured by small-angle X-ray scattering (SAXS) of similar elemental dusty plasmas^18,23,27,28^. Therefore, this plasma is characterized as a weakly-ionized, low-density dusty plasma, which contains components of the original basalt stone (e.g. silicon and iron oxides).

## Discussion

Modeling capabilities of volcanic phenomena are essential for both field- and laboratory-based studies. The contemporary research of volcanic processes incorporates a variety of theoretical and experimental models in an interdisciplinary approach, including thermodynamics, solid mechanics, fluid dynamics, and material sciences^29^. Theoretical models, e.g. of pyroclastic flows^30^, elastoplastic ground deformation^31^ and volcanic ash transport forecasting^32^, improve the understanding and the predictability of these processes. Laboratory models of volcanoes and lava phenomena employ analogues materials in order to experimentally study for instance lava domes^33^, submarine lava dynamics^34^ and mantle flows^35^.

In this interdisciplinary approach, the LMH-basalt study presented here may open new possibilities for experimental modeling in volcanology and geosciences. The controllable basalt core-melting and the lava-flow effects observed here in a miniature laboratory scale, and their resemblance to giant volcanic phenomena in nature, can be utilized for experimental small-scale simulations and demonstrations of various volcanic phenomena.

The natural ball-lightning phenomena, occasionally associated with volcanic eruptions, can also be experimentally modeled by the LMH-generated fire-column and fireball plasmas. A large number of fireball sightings has been recorded in the last centuries^22^, many occurring near volcanos or at times of heavy air pollution caused by volcanic eruptions. Spectrograph measurements of natural ball-lightning reveals the presence of Si, Fe, Ca and Al^36^ as in soil, in line with the theory suggesting that ball lightning is generated by lightning striking the soil^37^. These elements appear as well in the spectroscopy measurements of the basalt-generated plasma (along with other basalt components), in resemblance to the natural ball-lightning studies^22,36,37^. This finding adds another aspect to the visual similarity of microwave-generated fireballs to natural ball-lightning phenomena^2,38^).

The phenomena of lightning within eruptions of volcanic ash plumes in nature has also been recorded and studied^39^. This high-energy lightning chemically modifies and melts the volcanic ash^40^, generating spherical shapes similar to those observed in the powder accumulated in this study. For instance, the composition of the volcanic ash sampled in Mount St. Helens eruption on May 18, 1980^41^ includes SiO_2_ (65%), Al_2_O_3_ (18%), Fe_t_O_3_ (5%), and smaller amounts of MgO, CaO, Na_2_O, K_2_O, TiO_2_ and P_2_O. For comparison, the powder accumulated from the basalt-generated plasma consists of similar elements, as listed in Table 1. The relatively high concentration of phosphorous (and of Na and K as well) detected in the powder can be attributed to its relatively low melting temperature. This environmentally significant effect also features volcanic ash^42^.

Major challenges have remained for further research, including in-depth investigations and modeling of (a) the LMH interaction with basalt and phase transitions, (b) plasma ejection mechanisms and effects of thermionic emission, (c) microwave interactions with dusty plasma and particle agglomeration therein, (e) plasma-surface interactions, and plasma-chemical reactions occurring in this process.

In a more technology-oriented approach, this study of LMH in basalts could be relevant to various potential applications, including for instance (a) microwave-assisted drilling in basalts^20^, (b) cracking and crushing of basalts for mining and construction operations^43^, (c) basalt vitrification, glass production and coating, (d) joining of basalt bricks, (e) direct extraction of minerals in a powder forms by means of dusty-plasma production from basalts, and (f) analyses and identification of rock contents in the field by portable microwave-induced breakdown spectroscopy (MIBS) systems^44^ (as a variation of the laser-based LIBS technique^45^).

## Methods

### Method 1. Experimental

The processes of basalt melting and plasmoid ejection therefrom are initiated in this study by directing localized microwave power into the basalt specimen. This localization effect is implemented by the *microwave-drill* mechanism^20^, which creates the hotspot from which plasma is ejected in a form of a fire-column or a fireball, into the cavity.

The experimental setup shown in Fig. 2A,B includes a microwave cavity fed by a 2.45-GHz, 1-kW magnetron. The cavity is made of a 10 × 5 cm^2^ cross-section waveguide, shorted at the end by a mirror made of a periodic array of metallic vanes, under cutoff. Similar arrays are also installed as the sidewalls of the cavity. This cage structure prevents microwaves leaks, and yet enables a broad view into the waveguide with no disturbance to the various *in-situ* diagnostic means (such as the thermal camera and optical spectrometer which require direct lines of sight).

In this experiment we use basalt stones (from the Golan heights) either in their original natural shapes or cut to (3-cm)^3^ cubic bricks. This load is placed inside the cavity in an optimized position whereas the (optional) movable electrode directs the microwave energy locally into the substrate. Various level of focusing are optionally available in this setup, including (*i*) a single-electrode focusing as in Fig. 2A, (*ii*) no-electrode focusing, and (*iii*) a multi-electrode array focusing. Each electrode in this array expedites the intentional excitation of the hotspot in its vicinity, when brought into contact with the basalt-stone surface, and hence enables the stimulation of the LMH and fire-column (and consequently of a fireball) in a more controlled fashion. Typically, electrodes were applied to cubical bricks in these experiments, whereas no electrodes were used with natural stones.

The magnetron’s switched-mode power supply (MagDrive-1000, Dipolar Ltd., Sweden) provides an adjustable input microwave power up to 1 kW. The microwave power is delivered to the cavity via an isolator and an impedance auto-tuner (Homer, S-Team Ltd., Bratislava, Slovak republic), as depicted in Fig. 2B. The incident and reflected waves are recorded by the impedance-analyser mode of the auto-tuner, which also enables adaptive impedance matching, and optimal transmission of the microwave power into the cavity. The adaptive tuning is applied to intensify the hotspot heating in order to eject the plasma (whereas the synchronized de-embedded reflection coefficient measurements are presented and stored for offline analysis). In order to get more accurate measurements once the plasma is ejected, we turn off the adaptive tuning in order to measure the actual reflection coefficient instead of the de-embedded signal. This is possible due to the self-tuning effect of the plasmoid, as an alternative to the external impedance matching.

The scattered microwave is analysed in order to find the plasmoid impedance, and hence to estimate its dielectric and plasma properties. First, the reflections of the 2.45-GHz input power are measured during the various stages presented in Fig. 1A–D. The reflection measurements (as in Fig. 5C) enable us to find the impedance and dielectric properties of the fire-column by simulating an equivalent load, with the same shape, position and size.

The diagnostic means also include video recording, thermal imaging, optical emission spectroscopy (OES), and plasma *I-V* measurements, all conducted via the vane openings along the waveguide. A LabVIEW code controls the system, synchronizes the time between all the diagnostic means and accumulates the experimental data.

The video camera recording (at 200 fps) is used to monitor, record times and synchronize between effects such as inner hotspot formation, cracks, breakage, lava eruption and plasma ejection to other diagnostic means used in the experiment. Once ejected, the plasma column’s size, shape and position can be detected by the video recording. Also, evolution of fireballs, and their shape and movement are monitored by the video camera.

The FLIR-SC300 thermal camera monitors the spatial temperature evolution of the sample, the hotspot formation, and its temperature and size. The thermal imaging data is later compared to simulation results in order to validate the physical model.

The optical spectra emitted from the lava and plasma eruptions (Fig. 6A) are captured by an optical spectrometer (Avaspec-3648) with 0.3-nm resolution in the range 200–1000 nm. The results are analyzed for element identification and blackbody radiation, as well as radical emission curve fitting and Boltzmann plot (in order to find the rotational and excitation temperatures, respectively).

The excitation temperature is estimated, as in refs^18,23,27^, by using a Boltzmann plot of the line intensity, assuming a partial local thermal-equilibrium (pLTE) state of the plasma. The intensity *I*_*ki*_ of the transition from the upper *k* to the lower *i* energy level is given in the Boltzmann equation,

M1 $$\mathrm{ln}\,({I}_{ki}{\lambda }_{ki}/{g}_{k}{A}_{ki})=-\,(1/{k}_{B}{T}_{exc}){E}_{k}+{\rm{const}}.,$$

where *λ*_*ki*_ and *A*_*ki*_ are the transition wavelength and probability, respectively, *k*_*B*_ is the Boltzmann constant, and *E*_*k*_ is the upper energy state with a *g*_*k*_ degeneracy (as in ref.^23^, the relatively large scatter of the measurement results from the fit line in Fig. 6C is due to the 0.3-nm spectral resolution and the consequent overlap of the detected atomic lines).

A Langmuir-like probe is inserted into the plasma in order to measure its *I-V* characteristics. As shown in Fig. 7A(a), the 1.6-mm diameter, 50-mm long probe is fed by a 50-Hz, ~100-V alternating voltage. Valid results are only observed when the melt exists as illustrated in Fig. 7A(a), and closes the electric circuits to enable the current flow (otherwise much smaller currents are erroneously measured). A presumable reason is the isolating powder oxide accumulated on the metal surfaces, which adds a significant resistant to the plasma current loop. The hysteresis observed in the *I-V* curve may also indicate a contamination of the electrode, which leads to a capacitive effect.

The powder products are *ex-situ* examined, as in refs^18,23,27^, by a FEI Quanta 200FEG environmental SEM. The chemical element composition is analyzed using energy dispersive spectroscopy (EDS) with a Si(Li) liquid-nitrogen cooled Oxford INCA X-ray detector.

### Method 2: LMH model and simulation

The LMH process (prior to the plasma ejection) is modelled by a set of coupled EM-thermal equations^4,5^ for a time-varying (temperature-dependent) inhomogeneous lossy medium in a bounded region (a basalt stone in a cavity, in this case). The effective dielectric permittivity of the medium in the microwave regime is represented as a complex variable by

M2 $${\varepsilon }_{eff}(T)={\varepsilon }_{0}[{\varepsilon }_{r}^{{\rm{^{\prime} }}}(T)-j({\varepsilon }_{r}^{{\rm{^{\prime} }}{\rm{^{\prime} }}}(T)+{\sigma (T)/\omega \varepsilon }_{0})],$$

where *ε*_0_ is the permittivity of vacuum, *ε*′_*r*_ and *ε*″_*r*_ are the real and imaginary components, respectively, of the relative temperature-dependent permittivity of the medium (where *T*(*x*, *y*, *z*; *t*) is the temperature, spatially and temporally evolved during the process), and *σ*(*T*) is the temperature-dependent electric conductivity of the medium.

The EM-wave equation is given in the frequency domain by

M3 $$\nabla \times (\nabla \times \tilde{{\bf{E}}})-{\varepsilon }_{eff}(T){k}_{0}^{2}\tilde{{\bf{E}}}=0,$$

where $\tilde{{\bf{E}}}$ is the electric-field vector of the EM wave, *ω* is the angular frequency of the EM-wave, and ${k}_{0}=\sqrt{{\varepsilon }_{0}{\mu }_{0}}\omega$ is its wavenumber.

The heat equation is given in the time domain by

M4 $$\rho {c}_{p}\frac{\partial T}{\partial t}-\nabla \cdot ({k}_{th}\nabla T)=Q,$$

where *ρ* is the (local) medium density, *c*_*p*_ and *k*_*th*_ are its heat capacity and thermal conductivity, respectively, and *Q*(*x*, *y*, *z*; *t*) is the absorbed power density in the medium, as evolved during the LMH process. The latter is given by

M5 $$Q=\frac{1}{2}\omega {\varepsilon }_{0}{\varepsilon }_{r}^{{\rm{^{\prime} }}{\rm{^{\prime} }}}{|\mathop{{\bf{E}}}\limits^{ \sim }|}^{2}+\frac{1}{2}\sigma {|\mathop{{\bf{E}}}\limits^{ \sim }|}^{2},$$

for the dielectric and ohmic losses, respectively, and it couples the EM-wave and heat equations (Eqs M2 and M3, respectively, also coupled by the temperature dependence of the medium’s EM parameters). The single-frequency operation and the relatively slow-time variation of the temperature (in a > 1 ms scale) with respect to the EM-wave variation (in a ~1 ns time scale) enable us to apply a two-time scale approximation, hence to solve the EM-wave equation in the frequency domain and the heat equation in the time domain. The two solvers are iteratively applied, each with its relevant boundary conditions. The temperature-dependent parameters of basalt, employed in this analysis, are given with the relevant references in Table 2.

**Table 2:** The temperature-dependent basalt’s properties employed in the numerical simulations, *P*_*N*_(*p*_0_, *p*_1_, *p*_2_, … *p*_*N*_) denotes polynoms of the *N*-th order, ${\sum }_{n=0}^{N}{p}_{n}{T}^{n}$.

Property	Symbol	Unit	Value / Expression		Ref.
			*T* < *T*_*m*_	*T*_*m*_ ≤ *T* < 1,600 K	
Electric conductivity	*σ*(*T*)	Ω^−1^m^−1^	$110\cdot \exp (-12093/T)$	$3.8\cdot {10}^{5}\exp (-16977/T)$	^46^
Relative permittivity	*ε*_*r*_		8.18 - *j*0.708	5.95 − *j*0.208	^47, 48^
Mass density	*ρ*	kg⋅m^−3^	2,670	2,650	^49^
Specific heat	*c*_*p*_	J kg^−1^K^−1^	${P}_{4}(-217.4,\,6.4,-0.012,\,{10}^{-5},\,-3.1\cdot {10}^{-9})$	${P}_{3}(250.22,\,2.56,\,-0.003,\,1.26\cdot {10}^{-6})$	*
Thermal conduction	*k*_*th*_	Wm^−1^K^−1^	*P*3(0.4145, 0.0018, −9.2315⋅10^−7^, −1.1836⋅10^−10^)		^50^
Melting temperature	*T*_*m*_	K	1,360		^51^
Latent heat	*H*	J kg^−1^	40,000		^52^
Emissivity	${\varepsilon }_{s}$		0.74		^53^

^*^The specific heat is improted from the COMSOL-Multiphysics’ database.

Referring to the material properties essential for LMH, our previous microwave-drill analyses (e.g.^4^) show that the intentional-LMH effect is feasible in materials such as silicon, germanium, glass, and various ceramics (e.g. mullite, cordierite, zirconia, alumina of 86% purity, and clay) but not for instance in sapphire or pure alumina (due to their small dielectric losses). Also, the expansion coefficient and mass density may affect the material’s brittleness and cracking (which seem to appear in our basalt experiments after the inner melting). In glass, the optical transparency might be useful to visually observe the molten core inside, but it is not necessary for the hotspot evolution (the LMH effect may occur as well in opaque dielectric materials).

### Method 3. Dusty plasma interaction with microwaves

Following ref.^26^, the effective dielectric permittivity of the dusty plasma, *ε*_*r*_, consists of the plasma complex permittivity, ${\varepsilon }_{p}^{^{\prime} }$ − $j{\varepsilon }_{p}^{^{\prime\prime} }$, and of the dust conductivity *σ*_*ed*_^26^, as follows

M6a $${\varepsilon }_{r}={\varepsilon }_{p}^{{\rm{^{\prime} }}}-j{\varepsilon }_{p}^{{\rm{^{\prime} }}{\rm{^{\prime} }}}-j{\sigma }_{ed}/{\varepsilon }_{0}\omega ,$$

where *ε*_0_ is the vacuum permittivity and *ω* is the angular frequency. The dielectric permittivity of the plasma is given by its approximated real and imaginary components,

M6b $${\varepsilon }_{p}^{{\rm{^{\prime} }}}=1-\frac{{\omega }_{p}^{2}}{{\omega }^{2}+{\upsilon }^{2}},$$

M6c $${\varepsilon }_{p}^{{\rm{^{\prime} }}{\rm{^{\prime} }}}=\frac{{\omega }_{p}^{2}\upsilon }{\omega ({\omega }^{2}+{\upsilon }^{2})},$$

respectively, where ${\omega }_{p}=\sqrt{{e}^{2}{n}_{e}/{m}_{e}{\varepsilon }_{0}}$ and *υ* are the plasma and the collision frequencies, respectively, and *e*, *n*_*e*_ and *m*_*e*_ are the electron charge, density and mass, respectively. The dusty plasma conductivity is given in similar conditions by ref.^26^, as

M7 $${\sigma }_{ed}\cong {\eta }_{ed}\frac{\omega }{\hat{k}}[\frac{{\omega }^{2}-\upsilon {\upsilon }_{ch}}{({\omega }^{2}+{\upsilon }_{ch}^{2})({\omega }^{2}+{\upsilon }^{2})}+j\omega \frac{\upsilon +{\upsilon }_{ch}}{({\omega }^{2}+{\upsilon }_{ch}^{2})({\omega }^{2}+{\upsilon }^{2})}],$$

where *η*_*ed*_ is the charging factor, presented in terms of the dust collision length factor ${l}_{d}={({n}_{d}\pi {r}_{d}^{2})}^{-1}$, as ${\eta }_{ed}={\omega }_{p}^{2}{\varepsilon }_{0}/{l}_{d}$, with the dust grain density and the average particle radius denoted by *n*_*d*_ and *r*_*d*_, respectively. In this analysis, $\hat{k}$ represents the effective spatial angular frequency. The electron collision frequency is approximated by, *υ* = *V*_*Te*_*σ*_*n*_*N*_*n*_ where ${V}_{Te}=\sqrt{{k}_{B}{T}_{e}/{m}_{e}}$ is the electron thermal velocity, *σ*_*n*_ and *N*_*n*_ are the neutrals cross-section and density, respectively, *T*_*e*_ is the electron temperature, and *υ*_*ch*_ is the dust charging frequency.

As in refs^18,23^, the simulation here includes a loaded microwave cavity as shown in Fig. 2A. The plasma column inside is represented by a dielectric cylinder of *h* = 50mm height and *d*_*PC*_ = 15mm diameter. In the heuristic transmission-line model presented in ref.^23^, the plasma column is modeled by a lumped element in a transmission line (having admittances of *Y*_*PC*_ = *G*_*PC*_ + *jB*_*PC*_ and *Y*_*c*_, respectively). The conductance and susceptance of the plasma column are approximated by *G*_*PC*_ = *ωε*_0_${\varepsilon }_{r}^{^{\prime\prime} }$ *A*/*h* and *B*_*PC*_ = *ωε*_0_${\varepsilon }_{r}^{^{\prime} }$ *A*/*h*, respectively, where *A* is the effective cross-section area of the plasma-column. In this analysis (as in ref.^18^), the complex *ε*_*r*_ space is numerically scanned in order to find the conditions that provide reflection values as measured in the experiments. The real part of *ε*_*r*_ is initially chosen as 0.2 while the imaginary part is searched between 3 to 100. The simulation and the experimental observations, presented in the Smith chart in Fig. 5C(b), result in an estimate of ${\varepsilon }_{r}^{^{\prime\prime} }$ ~ 25 for the effective dissipation factor (including conductivity). Using the excitation temperature found by Boltzmann plot (~0.3–0.6 eV) and the dust particle size obtained by SAXS analyses of similar dusty plasmas^18,23,27,28^ with the assumptions therein (e.g., *υυ*_*ch*_ ≪ *ω*^2^ and *υ*_*ch*_ ≪ *ω*^2^), the dielectric constant and the corresponding electron density are estimated in this case by

M8 $${\varepsilon }_{r}\cong 1+\frac{{\omega }_{p}^{2}}{{\omega }^{2}+{\nu }^{2}}[-1+\frac{\nu }{\widehat{k}{l}_{d}}-j(\frac{\nu }{\omega }+\frac{1}{\widehat{k}{l}_{d}})],$$

and

M9 $${n}_{e}\sim \frac{{m}_{e}{\varepsilon }_{0}{\varepsilon }_{r}^{{\rm{^{\prime} }}{\rm{^{\prime} }}}}{{e}^{2}}\frac{{\omega }^{2}+{\upsilon }^{2}}{\upsilon /\omega +1/\hat{k}{l}_{d}},$$

respectively, where $\hat{k}$ is approximated by ~*π*/*d*_*PC*_ for the finite transverse profile of the plasma column. Equation (M9) yields *n*_*e*_ ~ 10^17^–10^18^m^−3^, similarly to other weakly-ionized plasmas and flames at atmospheric pressure in air.

When the dusty-plasma region coexists with the molten hotspot in the cavity, most of the microwave power is absorbed by the plasma. This domination is visually observed, since the molten rock tends to cool down once the plasma is ejected, and it is also evidenced by the thermal-camera measurements (e.g. in Fig. 7B(c)). This effect is also verified by simulation and by an analytical estimate (taking into account the effective values of the dielectric permittivity and electrical conductivity of both media, *σ*_eff_ = *σ* + *ωε*″ in a transmission-line model with lumped loads^23^). According to this estimate, the microwave power absorbed by the plasma column is more intense by at least one order of magnitude than the power absorbed then by the rock.

## Supplementary information

Supplementary Information – Description of video clips

Video S1a

Video S1b

Video S2

Video S3

Video S4

Video S5

Video S6

Video S7

## References

[1] Metaxas, A. C. *Foundations of Electroheat: A Unified Approach*. (John Wiley & Sons, 2000).

[2] Dikhtyar, V. & Jerby, E. Fireball ejection from a molten hot spot to air by localized microwaves. *Physical Review Letters* **96** (2006). 10.1103/PhysRevLett.96.045002 16486835

[3] Parris P, Kenkre V (1997). Thermal runaway in ceramics arising from the temperature dependence of the thermal conductivity. physica status solidi (b).

[4] Jerby E, Aktushev O, Dikhtyar V (2005). Theoretical analysis of the microwave-drill near-field localized heating effect. Journal of Applied Physics.

[5] Jerby E (2017). Localized microwave-heating intensification—A 1-D model and potential applications. Chemical Engineering and Processing: Process Intensification.

[6] Jerby, E., Dikhtyar V. & Einat, M. Microwave melting and drilling of basalt. *AIChE Annual Meeting*, Nov. 7–12, 2004, Austin, Texas, Proc., p. 1673, 2004 (Publisher: American Institute of Chemical Engineers).

[7] Satish H, Ouellet J, Raghavan V, Radziszewski P (2006). Investigating microwave assisted rock breakage for possible space mining applications. Mining Technology.

[8] Hartlieb P, Leindl M, Kuchar F, Antretter T, Moser P (2012). Damage of basalt induced by microwave irradiation. Minerals Engineering.

[9] Jerby, E., Meir, Y. & Faran, M. Basalt melting by localized-microwave thermal-runaway instability. *Proc. AMPERE 14th Int’l Conf. Microwave High Freq. Heat*., Sept. 16–19, 2013, Nottingham, UK, pp. 255–258 (2013).

[10] Hartlieb P, Kuchar F, Moser P, Kargl H, Restner U (2018). Reaction of different rock types to low-power (3.2 kW) microwave irradiation in a multimode cavity. Minerals Engineering.

[11] Hassani F, Nekoovaght PM, Gharib N (2016). The influence of microwave irradiation on rocks for microwave-assisted underground excavation. Journal of Rock Mechanics and Geotechnical Engineering.

[12] Mamontov AV, Nefedov VN, Tuv AL, Yazykov DA (2012). An investigation of the possibility of melting basalt using microwave energy. Measurement Techniques.

[13] Jerby, E. & Shoshani, Y. Fire-column-like dusty-plasma ejected from basalt by localized microwaves. *Proc., Microwave Discharges MD-10 Workshop*. Zvenigorod, Russia, Sept. 4–8 (2018).

[14] Savovic J (2017). LIBS Analysis of Geomaterials: Comparative Study of Basalt Plasma Induced by TEA CO2 and Nd:YAG Laser in Air at Atmospheric Pressure. Journal of Chemistry.

[15] Deák T, Czigány T (2009). Chemical Composition and Mechanical Properties of Basalt and Glass Fibers: A Comparison. Textile Research Journal.

[16] Ercenk E, Sen U, Yilmaz S (2012). The erosive wear behavior of basalt based glass and glass–ceramic coatings. Tribology International.

[17] Jerby, E. & Dikhtyar, V. Fireball ejection from a molten hotspot in solid to air by a reversed microwave drill mechanism. *Microwave Discharges: Fundamentals and Applications*. Lebedev, Y. A. (Ed.), Yanus-K, Moscow, 227–232 (2006).

[18] Meir Y (2013). Observations of ball-lightning-like plasmoids ejected from silicon by localized microwaves. Materials.

[19] Jerby, E. Microwave-generated fireballs. *Encyclopedia of Plasma**Technology*, J. L. Shohet (Ed.), CRC Press, 819–832 (2016).

[20] Jerby E, Dikhtyar V, Aktushev O, Grosglick U (2002). The microwave drill. Science.

[21] Lebedev YA, Epstein IL, Tatarinov AV, Shakhatov VA (2006). Electrode microwave discharge and plasma self-organization. Journal of Physics: Conference Series.

[22] Durand M, Wilson JG (2006). Ball lightning and fireballs during volcanic air pollution. Weather.

[23] Popescu S (2015). Plasma column and nano-powder generation from solid titanium by localized microwaves in air. Journal of Applied Physics.

[24] Kramida, A., Ralchenko, Y. & Reader, J. *NIST Atomic Spectra Database* (Ver. 5.2), NIST ASD Team (2014).

[25] Luque, J. & Crosley, D. R. *LIFBASE: Database and Spectral Simulation* (Version 1.5) (SRI International; Menlo Park, CA, USA: 1999).

[26] Shi Y-X, Ge D-B, Wu J (2007). Theoretical analysis of microwave attenuation constant of weakly ionized dusty plasma. Chinese Journal of Geophysics.

[27] Jerby E (2009). Nanoparticle plasma ejected directly from solid copper by localized microwaves. Applied Physics Letters.

[28] Mitchell, J. B. A. *et al*. Evidence for nanoparticles in microwave-generated fireballs observed by synchrotron x-ray scattering. *Physical Review Letters* **100** (2008). 10.1103/PhysRevLett.100.065001 18352481

[29] Fagents, S. A., Gregg, T. K. P. & Lopes, R. M. C. *Modeling Volcanic Processes the Physics and Mathematics of Volcanism*. (Cambridge University Press, 2013).

[30] Saucedo R, Macías J, Sheridan M, Bursik M, Komorowski J (2005). Modeling of pyroclastic flows of Colima Volcano, Mexico: implications for hazard assessment. Journal of Volcanology and Geothermal Research.

[31] Currenti G, Bonaccorso A, Negro CD, Scandura D, Boschi E (2010). Elasto-plastic modeling of volcano ground deformation. Earth and Planetary Science Letters.

[32] Heffter JL, Stunder BJB (1993). Volcanic ash forecast transport and dispersion (VAFTAD) model. Weather and Forecasting.

[33] Fink JH, Griffiths RW (1998). Morphology, eruption rates, and rheology of lava domes: Insights from laboratory models. Journal of Geophysical Research: Solid Earth.

[34] Gregg TKP, Fink JH (1995). Quantification of submarine lava-flow morphology through analog experiments. Geology.

[35] Funiciello F., Moroni M., Piromallo C., Faccenna C., Cenedese A., Bui H. A. (2006). Mapping mantle flow during retreating subduction: Laboratory models analyzed by feature tracking. Journal of Geophysical Research: Solid Earth.

[36] Cen, J., Yuan, P. & Xue, S. Observation of the optical and spectral characteristics of ball lightning. *Physical Review Letters* **112** (2014). 10.1103/PhysRevLett.112.035001 24484145

[37] Abrahamson J, Dinniss J (2000). Ball lightning caused by oxidation of nanoparticle networks from normal lightning strikes on soil. Nature.

[38] Ofuruton H, Kondo N, Kamogawa M, Aoki M, Ohtsuki Y-H (2001). Experimental conditions for ball lightning creation by using air gap discharge embedded in a microwave field. Journal of Geophysical Research: Atmospheres.

[39] Mcnutt SR, Williams ER (2010). Volcanic lightning: global observations and constraints on source mechanisms. Bulletin of Volcanology.

[40] Mueller, S. P., Helo, C., Keller, F., Taddeucci, J. & Castro, J. M. First experimental observations on melting and chemical modification of volcanic ash during lightning interaction. *Scientific Reports* **8** (2018). 10.1038/s41598-018-19608-3 PMC5780474 29362499

[41] Taylor HE, Lichte FE (1980). Chemical composition of Mount St. Helens volcanic ash. Geophysical Research Letters.

[42] Shoji S., Nanzyo M., Dahlgren R. (1993). Chapter 8 Productivity and Utilization of Volcanic Ash Soils. Developments in Soil Science.

[43] Buttress A (2017). Towards large scale microwave treatment of ores: Part 1 – Basis of design, construction and commissioning. Minerals Engineering.

[44] Meir Y, Jerby E (2011). Breakdown spectroscopy induced by localized microwaves for material identification. Microwave and Optical Technology Letters.

[45] Karatodorov S (2016). A novel device for spectrochemical analysis based on a combination of LIBS and a hollow cathode discharge. Journal of Physics: Conference Series.

[46] Presnall DC, Simmons CL, Porath H (1972). Changes in electrical conductivity of a synthetic basalt during melting. Journal of Geophysical Research.

[47] Frasch L, Mclean S, Olsen R (1998). Electromagnetic properties of dry and water saturated basalt rock, 1–110 GHz. IEEE Transactions on Geoscience and Remote Sensing.

[48] Carter, L. M. *et al*. Dielectric properties of lava flows west of Ascraeus Mons, Mars. *Geophysical Research Letters* **36** (2009).

[49] Bondre NR (2003). Analysis of vesicular basalts and lava emplacement processes for application as a paleobarometer/paleoaltimeter: a discussion. The Journal of Geology.

[50] Ebert HP (2002). Thermo-physical properties of a volcanic rock material. High Temperatures-High Pressures.

[51] Negro, C. D. *et al*. Lava flow hazards at Mount Etna: constraints imposed by eruptive history and numerical simulations. *Scientific Reports* **3** (2013). 10.1038/srep03493 PMC3861846 24336484

[52] Lange RA, Cashman KV, Navrotsky A (1994). Direct measurements of latent heat during crystallization and melting of a ugandite and an olivine basalt. Contributions to Mineralogy and Petrology.

[53] Burgi PY, Caillet M, Haefeli S (2002). Field temperature measurements at Erta’Alel lava lake, Ethiopia. Bulleting of Volcanology.

