# Supplementary material for: Localized microwave-heating (LMH) of basalt – Lava, dusty-plasma, and ball-lightning ejection by a “miniature volcano”
Source: Sci Rep. 2019 Sep 10;9:12954. doi: 10.1038/s41598-019-49049-5 (PMC6736850; doi:10.1038/s41598-019-49049-5)
Supplement: Supplementary file 1 — Supplementary Information – Description of video clips [file 41598_2019_49049_MOESM1_ESM.pdf]

Supplementary information

MS: SREP-18-49675

Title: Localized microwave-heating (LMH) of basalt – Lava, dusty-plasma, and ball-lightning ejection by a "miniature volcano"

Authors: Eli Jerby and Yoav Shoshani

Supplementary video clips:

1. (a) Lava erupting from the core of a basalt stone, flows downwards by gravity (while its visible color varies from luminous white to orange and red).
   (b) Lava erupting from a basalt stone, bubbling, and flowing downwards.
2. Lava eruption from two hotspots created by an electrode array in two opposite sides of the basalt brick. A lava tunneling effect in the core between the two hotspots is observed via the porous surface.
3. Plasma emission from a hotspot as a stable fire-column. Another plasmoid is also evolved in a form of a fireball alongside the fire-column.
4. A sole fireball is emitted from basalt by LMH and hovers in the air atmosphere within the cavity.
5. A fire-column emitted from a dome made of a thin layer of obsidian (blown up as a bubble by the lava eruption). This clip also shows a simultaneous ejection of two plasma columns from two different hotspots.
6. Variations in the complex reflection-coefficient (Γ), presented on a Smith-chart, show the adaptive feature of the microwave-generated plasmoid, which tends to maximize its power absorption (and hence to minimize reflections).
7. *I-V* characteristics measured by a single probe inserted into the plasma. Valid results are only observed when the melt flows down to the floor, and closes the electric circuits, hence enabling a current loop (smaller currents are measured otherwise due to the high impedance of the powder layer deposited by the plasma on the ceiling).
